# The laboratory in the multidisciplinary diagnosis of differences or disorders of sex development (DSD): I) Physiology, classification, approach, and methodology II) Biochemical and genetic markers in 46,XX DSD

**DOI:** 10.1515/almed-2021-0042

**Published:** 2021-07-08

**Authors:** Maria Luisa Granada, Laura Audí

**Affiliations:** Department of Clinical Biochemistry, Hospital Germans Trias i Pujol, Autonomous University of Barcelona, Badalona, Spain; Growth and Development Research Group, Vall d’Hebron Research Institute (VHIR), Center for Biomedical Research on Rare Diseases (CIBERER), Instituto de Salud Carlos III, Barcelona, Catalonia, Spain

**Keywords:** 46,XX DSD, biochemical diagnosis, differences/disorders of sex development (DSD), genetic diagnosis

## Abstract

**Objectives:**

The development of female or male sex characteristics occurs during fetal life, when the genetic, gonadal, and internal and external genital sex is determined (female or male). Any discordance among sex determination and differentiation stages results in differences/disorders of sex development (DSD), which are classified based on the sex chromosomes found on the karyotype.

**Content:**

This chapter addresses the physiological mechanisms that determine the development of female or male sex characteristics during fetal life, provides a general classification of DSD, and offers guidance for clinical, biochemical, and genetic diagnosis, which must be established by a multidisciplinary team. Biochemical studies should include general biochemistry, steroid and peptide hormone testing either at baseline or by stimulation testing. The genetic study should start with the determination of the karyotype, followed by a molecular study of the 46,XX or 46,XY karyotypes for the identification of candidate genes.

**Summary:**

46,XX DSD include an abnormal gonadal development (dysgenesis, ovotestes, or testes), an androgen excess (the most frequent) of fetal, fetoplacental, or maternal origin and an abnormal development of the internal genitalia. Biochemical and genetic markers are specific for each group.

**Outlook:**

Diagnosis of DSD requires the involvement of a multidisciplinary team coordinated by a clinician, including a service of biochemistry, clinical, and molecular genetic testing, radiology and imaging, and a service of pathological anatomy.

## I Physiology, classification, approach, and methodology

### 1) Physiology of sex differentiation and variant classification

Female and male sex characteristics are determined during fetal life by complex biological processes involving cascades of gene expression, which proteins have highly specific functions in location and time [1], [2], [3]. Genetic sex is established at conception, when a spermatozoon (with X or Y sex chromosome) fertilizes an oocyte (X chromosome), which results in a diploid 46,XX or 46,XY cell that determines the genetic sex. During the first weeks of embryonic life, sexually indifferent gonads and genitalia develop. The development of the urogenital sinus and the adreno–gonadal ridge occurs by the fourth week. The development of the bipotential gonad requires a cascade of gene expression (well characterized in humans: *EMX2, CBX2, NR5A1, GATA4,* and *WT1*) [1], [3].

**Figure 1:**
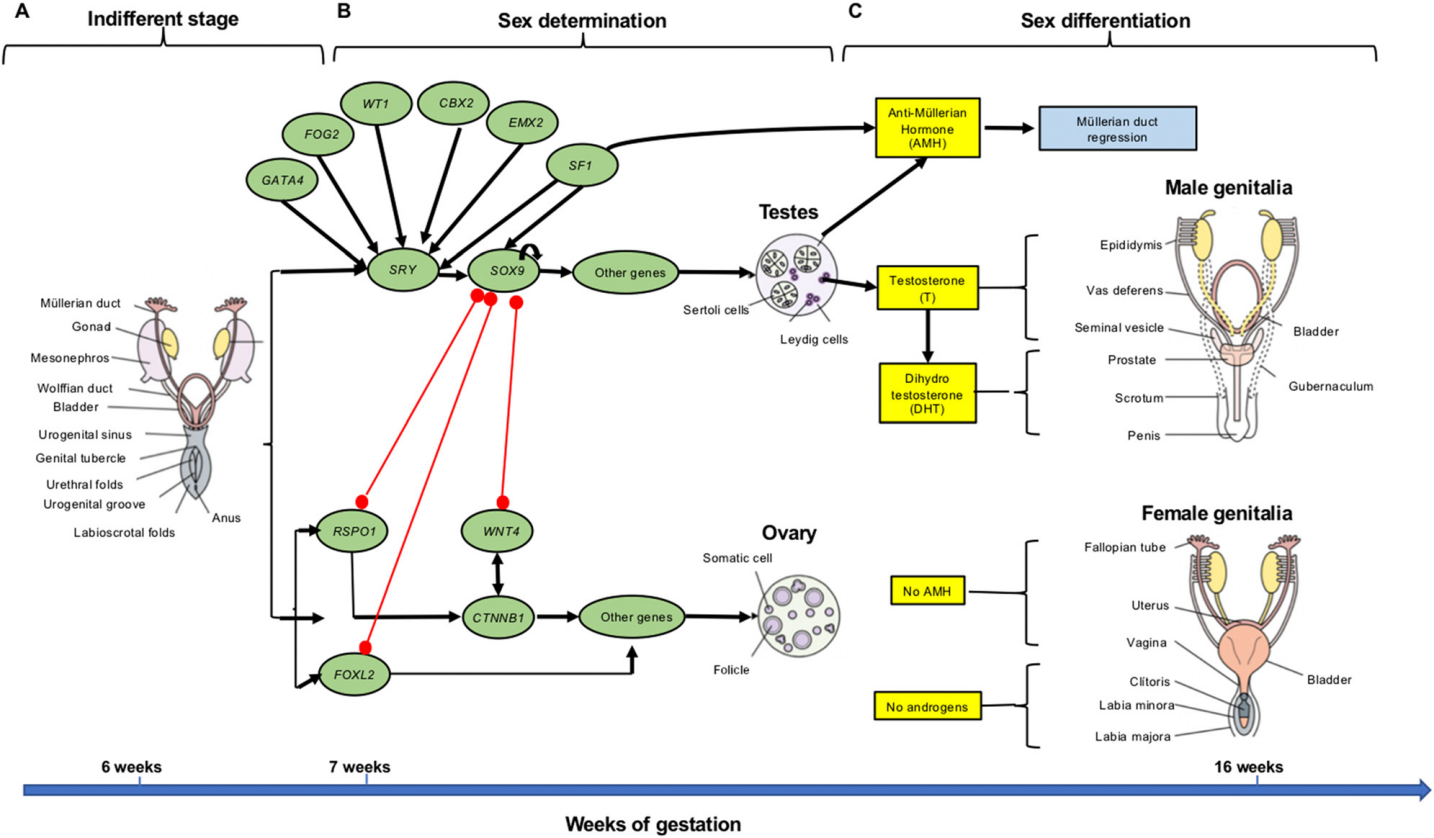
Human sex development during fetal life. (A) Indifferent stage: Bipotential gonads are developed by 5 weeks of life, as well as two pairs of genital ducts (Mullerian and Wolffian ducts), common external genitalia comprise the genital tubercle, the urethral folds, the urogenital groove and the labioscrotal folds. This stage ends by the sixth week. (B) Sex determination: Starts between sixth and seventh week when somatic cells and gonocytes in the bipotential gonad begin to differentiate into testicular or ovarian cells, depending on the presence and activation or repression of signaling pathways. Black arrows indicate gene activation whereas red ones indicate gene repression. (C) Sex differentiation: Internal and external genitalia differentiation depends on the presence or absence of testicular hormones (antiMullerian hormone [AMH] and testosterone [T] and dihydrotestosterone [DHT]) (with permission from ref. [16]).

From the sixth week, the presence of a Y chromosome and its *SRY* gene activates a cascade of genes that mediates the development of the undifferentiated gonad into a testis [4] and inhibits the expression of genes that induce gonad development into an ovary [1, 3, 5, 6]. Gonadal development is mediated by complex interactions between antagonic genes regulating processes that determine differentiation into a testis or an ovary [7] (Figure 1).

When gonads are sexually indifferent, male and female embryos share two genital ducts (Mullerian and Wolffian ducts) and external genitalia (genital tubercle and labioscrotal folds) [8]. Differentiation into internal and external genitalia depends on the secretion of specific hormones by the testes to an adequate amount following a specific timeline. Internal male genitalia require the secretion and action of testosterone (T), which causes Wolffian ducts to develop into the epididymis and vas deferens. The antiMullerian hormone (AMH) is also involved in this process, induces Mullerian duct regression and its activity is mediated by its receptor (AMHR2) [8]. The development of the prostate and external genitalia requires T metabolism, which is converted into dihydrotestosterone (DHT) by 5alpha-reductase type 2 [9] (Figure 1).

In the absence of AMH and elevated androgen concentrations (T and DHT), internal and external female genitalia develop (Figure 1). Although the murine model of estrogen receptor (ER) knockout suggests that estrogens could feminize the genital tubercle by mediation of ER [10], the morphology of the external genitalia of a female newborn with complete resistance to estradiol (E2) has not yet been described.

Any alteration along developmental stages may disrupt normal sex development, resulting in disorders/differences of sex development (DSD) [11], congenital conditions where the chromosomal, gonadal and/or genital development is atypical or different to the most frequent forms of development.

The Chicago Consensus [11] categorizes the causes of DSD according to the karyotype. Hence, DSD are categorized into three major groups: 1) Sex chromosome DSD, which occurs when the arrangement of sex chromosomes is different from the XX or XY pair; 2) 46,XX DSD with female karyotype, and 3) 46,XY DSD with male karyotype (Table 1). Each group is divided into subgroups (Table 1). In Groups 2 and 3, a broad range of genes are involved, which increase over the years.

**Table 1:** Classification of disorders or differences of sex development (DSD) based on the sex chromosomes present in the karyotype [11].

1) Sex chromosome DSD	
1. 47,XXY: Klinefelter syndrome and variants 2. 45,X0 and 45,X0/46,XX mosaicism (Turner syndrome and variants) 3. 45,X0/46,XY mosaicism (mixed gonadal dysgenesis) 4. 46,XX/46,XY mosaicism (ovotesticular DSD) 5. 47,XYY	
2) 46,XX DSD	
1. Abnormal gonadal development	a. Partial (PGD) or complete (CGD) gonadal dysgenesis b. Ovotesticular DSD c. Testicular DSD
2. Abnormal genital development for androgen excess	**Fetal production:** a. 21 hydroxylase deficiency b. 3β- hydroxy steroid deficiency c. 11-β hydroxylase deficiency d. Glucocorticoid resistance e. Estrogen resistance
	**Feto-placental production:** a. P450-oxidoreductase deficiency b. Placental and fetal aromatase deficiency c. Androgen secreting fetal or placental tumors
	**Material origin:** a. Therapeutic agents or environmental pollutants b. Maternal CAH c. Maternal virilizing tumors (luteomas, Krukenberg tumor)
3. Abnormal development of internal genitalia	a. Hand–foot–genital syndrome b. MURCS syndrome (Mullerian aplasia, renal aplasia, cervico–thoracic eomite abnormalities) c. MRKH syndrome (Mayer–Rokitansky–Kuster–Hauser), types I and II
3) 46,XY DSD	
1. Abnormal gonadal development	a. Partial (PGD) or complete (CGD) gonadal dysgenesis b. Ovotesticular DSD c. Ovarian DSD
2. Abnormal genital development secondary to deficient androgen synthesis or action	**Disorders of androgen synthesis:** a. Insensitivity to LH (Leydig cell aplasia/hypoplasia) b. 7-dehydrocholesterol reductase deficiency (Smith–Lemli–Opitz syndrome) c. Star protein deficiency (lipoid congenital adrenal hyperplasia) d. Cholesterol desmolase deficiency e. 3β-hydroxy steroid dehydrogenase type 2 deficiency f. 17α-hydroxylase/17–20 desmolase deficiency g. P450-oxidoreductase deficiency h. Cytochrome *B*5 deficiency i. Defective backdoor pathway adrenal steroidogenesis j. 17β-hydroxy steroid dehydrogenase type 3 deficiency k. 5α-reductase type 2 deficiency l. Isolated hypospadias and/or cryptorchidism
	**Disorders of androgen action**: a. Complete or partial androgen insensitivity b. Therapeutic agents or environmental pollutants
3. Abnormal genital development secondary to defective anti-Müllerian (AMH) hormone synthesis or action	**Persistent Müller ducts**: a. AntiMullerian hormone deficiency b. Resistance to antiMullerian hormone
4. Complex malformation syndromes	a. Malformative syndromes with abnormal genital development (cloacal malformations, Aarskog syndrome, Robinow syndrome, among others) b. Severe, early-onset, intrauterine growth restriction

DSD, disorder or different sex development; CAH, congenital adrenal hyperplasia.

In Group 1, sex chromosome DSD is defined by the number or arrangement of sex chromosomes (Table 1). The most frequent are: 47,XXY (Klinefelter syndrome); 45,X0 (Turner syndrome) and its variants, including 45,X/46,XY mosaicism (or mixed gonadal dysgenesis); 46,XX/46,XY mosaicism (true sex chromosome chimerism or ovotesticular DSD); and 47,XYY.

Group 2, with 46,XX DSD karyotype (Table 1), includes: 1) Gonadal development disorders (CGD, PGD, ovotesticular chimerism [ovotesticular DSD], or testicular development [testicular DSD]); 2) genital development disorders caused by exposure to excess androgen levels (from fetal, fetoplacental or maternal origin) that cause the virilization of external genitalia; and 3) internal genitalia development disorders.

Group 3, with 46,XY karyotype (Table 1), includes: 1) Gonadal development disorders ([CGD, PGD, ovotesticular development [ovotesticular DSD], or ovarian development [ovarian DSD]); 2) disorders of androgen synthesis or action; 3) disorders of AMH synthesis or action; and 4) complex malformative syndromes affecting the development of the genitourinary and digestive system and severe early-onset intrauterine growth retardation, which is associated with hypospadias.

With the exception of Group 1, sex chromosome DSD (especially, Klinefelter syndrome with 47,XXY karyotype), and Group 3, 46,XY DSD male infants born with congenital hypospadias, the population frequency of Group 2 and 3 DSD is so low that they are considered “rare diseases” (population frequency <1/2,000).

### 2) Multidisciplinary teams for DSD diagnosis

DSD manifests either at birth or early after birth in the form of ambiguous external genitalia, by discordance between prenatal karyotype and genital development, a family history of DSD, concurrent acute adrenal insufficiency, or when the presence of a gonad is detected in an inguinal hernia. Later in life, during puberty, DSD is identified by discordances between gonadal and genital development. In addition, undiagnosed adults might seek medical advice for infertility or other health problems such as arterial hypertension. Studies to investigate the etiology of these problems may lead to the detection of a DSD.

Diagnosis of the cause of a DSD is challenging and will depend on the knowledge and skills of each specialist involved, added to the performance of the multidisciplinary team [11], [12]. All protocols emphasize that DSD diagnosis requires the involvement of a multidisciplinary team coordinated by a clinician [12], [13] that includes a Service of Biochemistry (general biochemistry and specific markers or hormones); a Service of Clinical and Molecular Genetics (initial karyotype and interpretation of the results of other studies will guide further studies); a Service of Radiology and Imaging (pelvic ultrasonography to detect internal genital structures and the presence of intraabdominal gonads); and a Service of Anatomic Pathology (when analysis of gonad structure is required).

A variety of diagnostic algorithms have been designed [13], [14], [15], [16], which have evolved as new technologies emerged, mainly in the fields of imaging, biochemistry and, most importantly, molecular diagnostics [17], [18], [19], [20], [21].

### 3) Biochemical and genetic studies for the diagnosis of DSD

#### a) Basal biochemical studies

Biochemical tests and, especially, hormone determinations, play a crucial role in initial diagnosis of DSD, follow-up, and monitoring of response to treatment. There are two major groups of hormones: steroid and peptide hormones.

**Figure 2:**
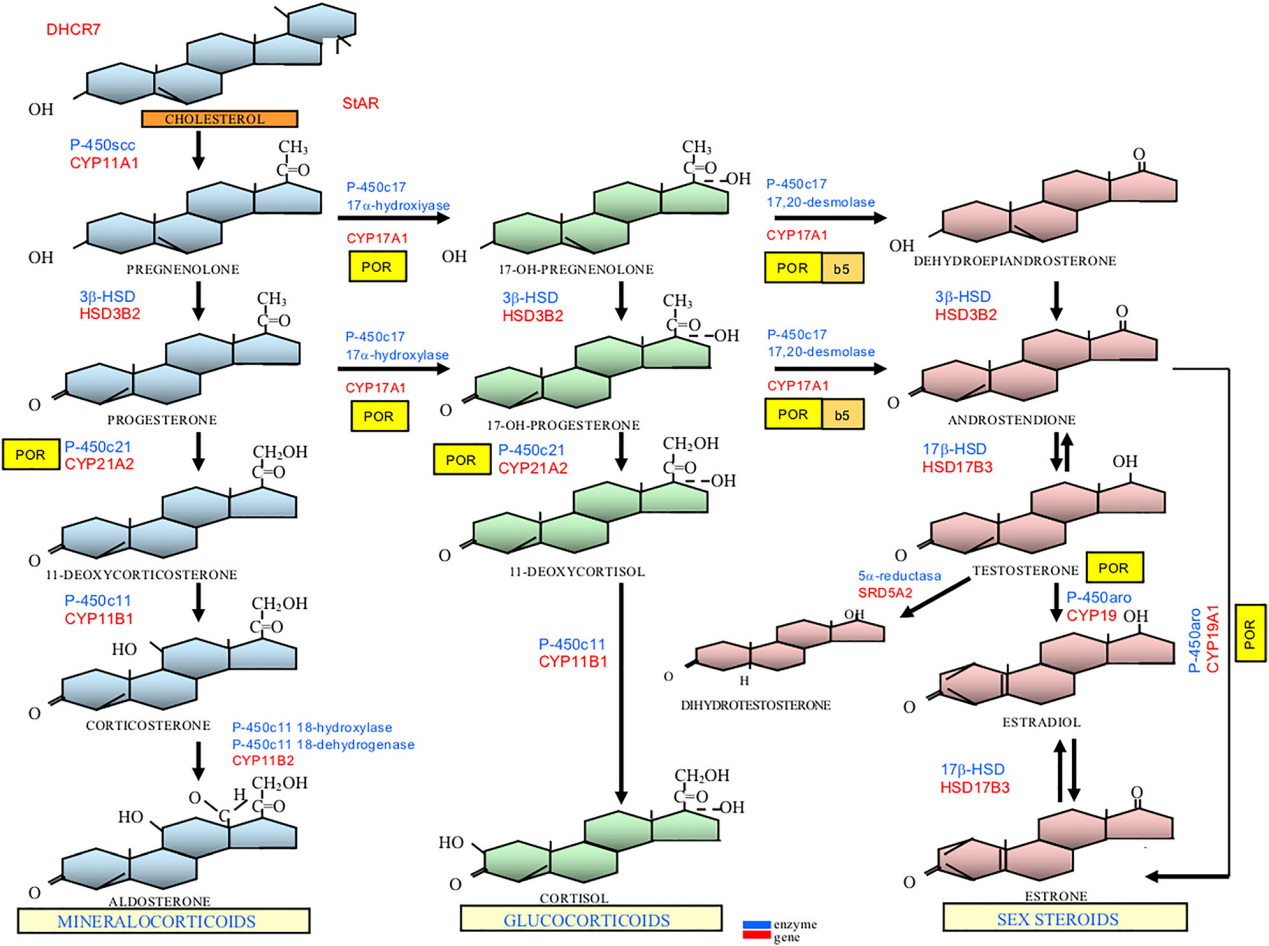
Adrenal and gonadal steroidogenesis. In the adrenal glands, biosynthesis progresses from cholesterol to cortisol (glucocorticoid pathway) and to aldosterone (mineralocorticoid pathway). In the gonads, precursors progress to sex steroids: testosterone (T) as main androgen and estradiol as main estrogen. T is peripherally metabolized into dihydrotestosterone (DHT) as the most active androgen. In blue: Enzyme abbreviations; in red: Enzyme coding gene abbreviations; yellow square: Coenzyme POR (P450-oxidoreductase); beige square: Cytochrome *b*5. DHCR7, *DHCR7* gene (7alpha-dehydrocholesterol reductase); StAR, *steroid acute regulatory protein* (*StAR* gene); P-450scc, P-450 *side-chain cleavage*, cholesterol desmolase (*CYP11A1* gene), 3β-HSD, 3β-hydroxysteroid dehydrogenase type 2 (*HSD3B2* gene); P-450c17, 17α-hydroxylase/17,20-desmolase or lyase (*CYP17A1* gene); P-450c21, 21-hydroxylase (*CYP21A2* gene); P-450c11, 11β-hydroxylase type 1 (*CYP11B1* gene); P-450c11 18-hydroxylase [corticosterone methyl-oxidase type 1 (CMO-I)] and P-450c11 18-dehydrogenase [corticosterone methyl-oxidase type II (CMO-II)] (*CYP11B2* gene); 17β-HSD, 17β-hydroxysteroid dehydrogenase type 3 or 17-ketoreductase (*HSD17B3* gene), 5α-reductase type 2 (*SRD5A2* gene); and P-450aro, aromatase (*CYP19A1* gene).

Steroid hormones (Figure 2) are synthetized from cholesterol in the adrenal cortex, the gonads, and the placenta, although they are metabolized in numerous peripheral tissues. The methods for steroid hormone determination in blood and urine have evolved over time to the current use of immunoassay and mass spectrometry. There are commercially available immunoassays for the steroids most frequently measured in routine practice. However, determination of other parameters of interest in the diagnosis of DSD such as corticosterone, deoxycorticosterone, 17-OH-pregnenolone, and DHT requires the use of liquid chromatography–mass spectrometry (LC–MS/MS) [22], [23]. International scientific societies recommend the use of mass spectrometry-based methods (LC–MS/MS and gas chromatography–mass spectrometry [GC–MS/MS]) for measuring sex steroids and their precursors in the diagnosis of DSD, especially, in neonates [24]. These methods measure different steroids in the same sample, including metabolites that cannot be determined by specific immunoassays [25]. Steroids can be measured in different samples: serum, blood, saliva, and urine [26]. It is very important that the laboratory meets quality standards, is involved in external quality assurance programs, and establishes specific age- and sex-specific reference intervals [24]. Given that adrenal steroids have a marked circadian rhythm, it is recommended that determination is performed early in the morning (8–9 a.m.).

Peptide hormones are determined by noncompetitive immunoassays, which have a high sensitivity but which specificity is not always known. The lack of gold-standard methods and inconsistent standardization hinder inter-assay comparison. Thus, age- and sex-specific reference values are required for each assay [27], [28]. Peptide hormones related to sex development have a marked sexual dimorphism. In the first years of life of male infants, luteinizing hormone (LH) concentrations are higher than those of follicle-stimulating hormone (FSH), and the LH/FSH ratio is clearly higher [29]. AMH and inhibin B (INHB) concentrations are 100 times and 10 times higher, respectively, in males than in females. A guide was recently published for peptide hormone testing for the diagnosis of DSD [30].

#### b) Functional tests

In some cases, basal hormone testing is not informative enough and stimulation tests are required to identify secretion deficiencies.

There are three types of functional tests:

##### b-1) Adrenocorticotropic hormone (ACTH) stimulation test [31]

This test is used to investigate adrenal steroidogenesis (Figure 2). Stimulation is induced by endovenous administration of synthetic ACTH (1–24) (Cosyntropin or Synacthen^®^) at a dose of 0.25 mg (in infants, it can be reduced to 0.125 mg). Adrenal enzyme deficiencies are investigated through determination of a range of hormones and precursors at baseline and at 60 min from stimulation [32].

##### b-2) Chorionic gonadotropin (HCG) stimulation test [33]

HCG that binds the LH-CG receptor of Leydig cells stimulates the production of testicular androgens. Different stimulation protocols have been developed. Determination of androgens and their precursors is performed before and at 48–72 h after the last injection. For diagnosis of DSD, it is important that T, its precursors, and its DHT metabolite are measured to identify enzyme deficiencies in testicular and peripheral steroidogenesis (Figure 2).

##### b-3) Gonadoliberin (GnRH) stimulation test

Stimulation can be induced using the gonadotropin (LH and FSH) releasing hypothalamic factor (gonadoliberin or GnRH) **(**Luforan^®^ 100 µg i.v and 25–50 µg in children), or most recently, using GnRH analogs (leuprorelin acetate [Procrin^®^], buserelin acetate). A LH response >5 IU/L indicates the central activation of the hypothalamic–pituitary–gonadal axis (HPG) [34], [35], [36].

#### c) Genetic testing

##### c-1) Cytogenetics and karyotype

The karyotype is essential for DSD categorization into one of the three diagnostic groups based on the sex chromosomes found (Table 1). The gold-standard method is cytogenetics, although array-complementary genomic hybridization (array-CGH) techniques are increasingly used [21].

Apart from alterations in sex chromosomes, some DSD may involve copy number variations (CNV) (deletions, duplications, translocations), both in autosomes and sex chromosomes, which is especially relevant when the phenotype includes additional anomalies to DSD [37], [38], [39], [40]. CNV is detected by array-CGH and can be detected by karyotype determination by array-CGH.

##### c-2) Genetic testing

The most frequent monogenic causes of DSD were identified in the late 20th century with the cloning of the genes codifying proteins that were known to be altered in the clinical and biochemical phenotype. This was especially useful for determination of enzyme deficiencies in adrenal and gonadal steroidogenesis (Figure 2), both in 46,XX and 46,XY DSD, and in complete androgen insensitivity. In contrast, the genes involved in the differentiation and development of male and female gonads are being progressively detected on the basis of family studies, animal models and functional studies *in vitro* [7], [41]. A large number of genes involved in the development of DSD encode the regulatory transcription factors of other genes (i.e., *AR, DAX1, DMRT1, FOXL2, NR5A1, SOX3, SOX9*, and *SRY*). The literature demonstrates the presence of mutations in noncoding regulatory regions, which suggests that testing noncoding DNA regions will be useful to identify the cause of some DSD in which molecular diagnosis could not be previously performed [42].

Structural analysis of a specific candidate gene is performed by automated Sanger DNA sequencing, which involves PCR amplification of coding and flanking regions and, eventually, of the promoter region. However, the introduction of high-throughput DNA sequencing allows whole exome testing (coding regions) or screening a panel of candidate genes. In addition, broad expressivity in some DSD phenotypes could be explained by an oligogenic origin, in which interaction of multiple genes could give rise to a phenotype unique to each individual [43]. The availability of these techniques in genetic testing laboratories has increased significantly as a result of improvements in their quality and cost. Thus, testing a specific gene or one of its regions will progressively be limited to the diagnosis of a new patient who is a relative of a well characterized case [21, 44–49].

## II Biochemical and genetic markers in 46,XX DSD

Table 2 contains a list of monogenic causes of DSD in the 46,XX karyotype group. The list is progressively enriched over time, especially in relation to the causes of dysgenetic gonadal development.

**Table 2:** Clinical diagnoses and genes involved in disorders or differences of sex development (DSD) of monogenic etiology.

DSD with 46,XX karyotype		
Clinical diagnosis	Gene (locus)	OMIM (inheritance) (additional phenotype)
**1. 46,XX DSD secondary to impaired gonadal development: gonadal dysgenesis, ovotesticular DSD, testicular DSD**		

Gonadal dysgenesis	*BMP15* (Xp11.22)	300510/300247 (D)
Gonadal dysgenesis	*ESR2* (14q23.2-q23.3)	618187 (DA)
Testicular DSD	*FGF9* (13q12.11)	600921 (DA:dup) (only one case reported)
Gonadal dysgenesis	*FOXL2* (3q22.3)	608996 (DA) (Blepharophimosis, epicanthus inversus and ptosis, types I and II)
Gonadal dysgenesis	*MYRF (11q12.2)*	608329 (DA) 618280 (DA) (cardiac–urogenital syndrome)
Testicular DSD	*NR2F2 (15q26.2*	615779 (DA) (congenital heart disease, diaphragmatic hernia, blepharophimosis, ptosis, and epicanthus inversus syndrome)
1) Gonadal dysgenesis 2) Ovotesticular DSD 3) Testicular DSD	1) *NR5A1* (9q33.3) 2) *NR5A1* (9q33.3) (p.Arg92Trp) 3) *NR5A1* (p.Arg92Trp)	612964 (DA) 617480 (DA)
Gonadal dysgenesis	*NUP107* (12q15)	607617 (RA) (reported in a consanguineous family; other phenotypes with nephrotic syndrome)
Ovotesticular DSD	*RSPO1* (1p34.3)	610644 (RA) (palmoplantar keratodermas and squamous-cell skin carcinoma)
1) Ovotesticular DSD 2) Testicular DSD	*SOX3* (Xq27.1)	313430 (XL:dup)
Gonadal dysgenesis	*SOX8* (16p13.3)	605923 (primary ovarian insufficiency)
1) Ovotesticular DSD 2) Testicular DSD	*SOX9* (17q24.3)	278850 (DA:dup)
1) Ovotesticular DSD 2) Testicular DSD	*SOX10* (22q13.1)	609136 (DA:dup) (Waardenberg and Hirschsprung syndromes, peripheral neuropathy)
1) Ovotesticular DSD 2) Testicular DSD	*SRY* (Yp11.2)	400045 (T)
1) Ovotesticular DSD 2) Testicular DSD	*WNT4* (1p36.12)	158330 (DA) 611812 (RA): SERKAL syndrome (sex reversal dysgenesis of kidneys, adrenals, and lung), lethal biallelic
1) Ovotesticular DSD 2) Testicular DSD	*WT1* (11p.13)	DA

**2. 46,XX DSD with normal gonadal but abnormal genital development due to excess of fetal or fetoplacental androgens**		

CAH due to 21-hydroxylase deficiency	*CYP21A2* (6p21.33)	201910 (RA) (adrenal deficiency)
CAH due to 3β-hydroxy steroid dehydrogenase type 2 deficiency	*HSD3B2* (1p12)	201810 (RA) (adrenal and gonadal deficiency)
CAH due to 11β-hydroxylase deficiency	*CYP11B1* (8q24.3)	202010 (RA) (adrenal deficiency)
Insensitivity to glucocorticoids	*GRα (NR3C1)* 5q31.3	615962 (DA) (hypertension)
Insensitivity to estrogens	*ESR1* (6q25.1-q25.2)	615363 (RA) (overgrowth, osteoporosis, polycystic ovary) (only one case reported)
P450-oxidoreductase deficiency	*POR* (7q11.23)	201750 (RA) (17α-hydroxylase deficiency, variable deficiencies of 21-hydroxylase and aromatase) (Antley-Bixler syndrome, ±craniosynostosis)
Aromatase deficiency	*CYP19A1* (15q21.2)	613546 (RA) (maternal and fetal virilization)

**3. 46,XX DSD with normal gonadal but abnormal Mullerian duct development**		

Hand–foot–genital syndrome	*HOXA13* (7p15.2)	140000 (DA)
MURCS syndrome (Mullerian aplasia, renal aplasia, cervico–thoracic Somite abnormalities) MRKH syndrome (Mayer–Rokitansky–Kuster–Hauser), types I and II Müllerian aplasia and hyperandrogenism	Multigenic: Del 17q12 CNV on 17q12, 1q21.1, 22q11.21, Xq21.31 Dupl *SHOX* *WNT4* (1p36.12)	601076 277000/614527/267400/192050 158330 (DA)

DSD, disorder or different sex development; CAH, congenital adrenal hyperplasia; D, dominant; DA, dominant autosomal; RA, recessive autosomal; XL, X-linked; T, translocation; Dup, duplication; Del, deletion; CNV, copy number variation.

### 1) Abnormal gonadal development

Gonadal development disorders with 46,XX karyotype include PGD and CGD, ovotesticular gonads (ovotesticular DSD) and testis development (testicular DSD) (Table 1).

The most frequent are PGD and CGD. None of these disorders is associated with genital ambiguity, and individuals have a female phenotype at birth. Clinical manifestations include delayed and/or absent puberty. Biochemical markers show elevated levels of LH and FSH, undetectable AMH and prepubertal estradiol (E2) concentrations. Other precursors such as androstenedione, 17-α-hydroxyprogesterone (17OH-P), and T also are at prepubertal concentrations, whereas dehydroepiandrosterone (DHEA) and its sulphate increase during normal adrenarche. A milder and relatively frequent clinical form is early menopause or early ovarian failure, in which biochemistry shows an early increase in LH and FSH concentrations, reduced levels of AMH (a good marker of ovarian reserve), and low levels of E2 and progesterone (P). Evidence is progressively published on monogenic causes (Table 2), among them, inactivating mutations in *BMP15, ESR2, FOXL2, MYRF, NR5A1, NUP107*, and *SOX8* genes*.* In most cases, the effect is dominant (except for *NUP107*) and, in some cases, they are associated with other phenotypic characteristics (Table 2).

Ovotesticular or testicular development shows genital ambiguity (even fully male external genitalia) from birth, due to fetal exposure to elevated levels of T. The biochemical profile in newborn or infant is similar to that observed in males with 46,XY karyotype. During childhood, the ability of the gonads to produce T can be assessed by HCG testing. During puberty, there is an increase in T concentrations that does not reach normal male concentrations, resulting in elevated levels of LH and FSH. Most monogenic causes give rise either to an ovotesticular or testicular DSD (Table 2). The first monogenic cause to be identified was the translocation of a fragment of the Y chromosome containing the *SRY* gene to an autosome. Some monogenic causes are associated with complex phenotypes such as mutations in *NR2F2, RSPO1, SOX10, WNT4* and, more recently, *WT1*; in the case of the *NR5A1* gene, only the p.Arg92Trp mutation causes ovotesticular or testicular development; duplications in the *FGF9, SOX3,* and *SOX9* genes have also been described.

### 2) Disorders of genital development due to androgen excess

When the gonads differentiate into ovaries and the internal genitalia are female, fetal exposure to elevated levels of androgens causes the virilization of the external genitalia. The origin of these androgens may be fetal, fetoplacental, or maternal (Table 1).

#### a) Increased androgens of fetal origin

Virilization of the external genitalia in most newborns with the 46,XX karyotype is induced by congenital adrenal hyperplasia (CAH). The most frequent cause is 21-hydroxylase deficiency [32], [50] (*CYP21A2* gene). Its “simple virilizing” form is associated with cortisol deficiency, ACTH elevation, and the accumulation of 17OH-P, androstendione, and T (Figure 2). In the most severe forms, also known as “salt-wasting adrenogenital syndrome”, it is associated with aldosterone deficiency with concurrent hyponatremia, hyperkalemia, and increased plasma renin activity (PRA). Biochemical diagnosis is based on the finding of elevated levels of 17OH-P (basal or post ACTH >300 nmol/L [>10,000 ng/dL]), androstenedione, and T. Determination of glucose and electrolytes in blood and PRA is also necessary [51]. It is important to establish reference values for gestational age, since preterm newborns show significantly higher 17OH-P concentrations, which results in high rates of false positives [52]. Determination of 21-deoxycortisol originated from 17OHP conversion by 11β-hydroxylase can be useful to minimize false positives, since it is elevated in the presence of 21-hydroxylase deficiency, but not in other adrenal deficiencies or in preterm newborns; its determination, however, is not available in many clinical laboratories [53].

**Figure 3:**
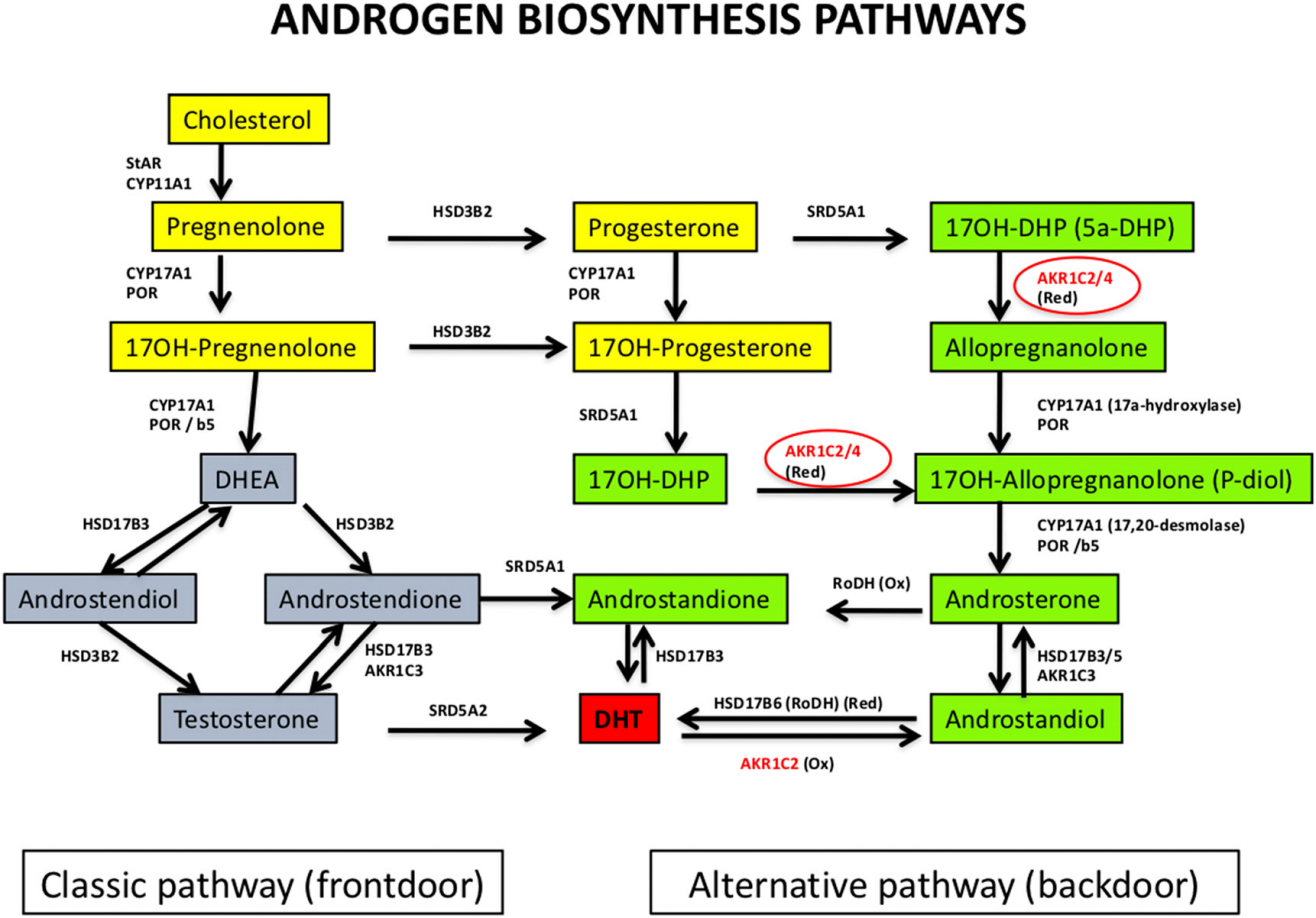
Androgen biosynthesis pathways: classic pathway (frontdoor) and alternative pathway (backdoor). Classic pathway progresses from cholesterol to testosterone (T) by mediation of the pregnenolone [StAR protein (*STAR* gene) and cholesterol desmolase enzyme (*CYP11A1* gene)], 17-OH-pregnenolone [17alpha-hydroxylase enzyme (*CYP17A1* gene) and coenzyme P450-oxidoreductase (*POR* gene)], dehydroepiandrosterone (DHEA) [17,20-desmolase enzyme (*CYP17A1* gene), coenzyme P450-oxidoreductase (*POR* gene) and cytochrome *b*5 (*CYB5A* gene)], androstenedione or androstenediol [enzymes 3beta-hydroxysteroid dehydrogenase type 2 (*HSD3B2* gene), 17beta-hydroxysteroid dehydrogenase type 3 (*HSD17B3* gene), and aldo-keto reductase family 1 member C3 (*AKR1C3* gene)]. T is transformed into dihydrotestosterone (DHT) by the enzyme 5alpha-reductase type 2 (*SRD5A2* gene). DHT can also be converted into androstanedione by androstenedione metabolism [enzyme 5alpha-reductase type 1 (*SRD5A1* gene)] and to DHT [enzyme 17β-hydroxysteroid dehydrogenase type 3 (*HSD17B3* gene)]. Alternative pathway synthesizes DHT overcoming T synthesis. Progesterone (product of pregnenolone) and 17OH-progesterone (product of progesterone) are transformed into 17OH-DHP (5alpha-dihydroxy-progesterone). The latter is transformed into androstanedione or androstanediol which, in turn, are metabolized into DHT [acting several previously described enzymes as well as aldo-keto reductase family 1 members C2 y C4 (*AKR1C2* y *AKR1C4* genes)*,* retinol-dehydrogenase (*RODH* gene), 17beta-hydroxysteroid dehydrogenase type 5 and type 6 (*HSD17B5* y *HSD17B6* genes)]. (Ox), oxidation; (Red), reduction.

Urine steroid determination by GC–MS/MS shows the activation of alternative backdoor pathway (Figure 3), concomitant to elevated levels of 5α-pregnane-3α,17α-diol-20-one (P-diol), pregnanetriol (P-triol), 17OH-pregnanolone, and an elevation of the androsterone/ethiocholanolone ratio [54].

There are mild forms of this enzyme deficiency, known as “nonclassical” or “late-onset”, which manifest as early pubarche, with a slight acceleration of growth velocity and bone maturation, and the appearance of pubic hair. As to the biochemical profile, there is a slight increase of basal 17OH-P with or without androstenedione and T elevation. ACTH stimulation will reveal excess 17OH-P (31–300 nmol/L; 1,000–10,000 ng/dL).

Molecular diagnosis will confirm the presence of mutations in the *CYP21A2* gene in homozygosity or in compound heterozygosity, the effect of which is the total or almost-total inactivation of enzyme activity in the most severe forms with concomitant salt loss, as well as in the simple virilizing forms. There is an association between the genotype and the degree of virilization of patients with typical forms of the enzyme deficiency [55]. In nonclassical or late-onset forms, one of the alleles may carry a mild-effect mutation that would allow some enzymatic activity. Mutations in the *CYP21A2* gene are the most frequent molecular disorder in humans, and its incidence varies as a function of the geographical region and social structure [32], [56] (Table 2).

Other less frequent causes of CAH are 3β-hydroxy-steroid dehydrogenase type 2 (3βHSD2), 11β-hydroxylase, and cytochrome P-450 oxidoreductase (POR) deficiencies [57].

The enzyme 3βHSD2 (*HSD3B2* gene) catalyzes two sequential reactions converting pregnenolone into P, 17OH-pregnenolone into 17OH-P, and DHEA into androstendione (Figure 2). Patients with severe deficiency have CAH secondary to impaired cortisol and aldosterone synthesis. Patients exhibit elevated levels of steroids Δ5 (pregnenolone, 17OH-pregnenolone, DHEA) and a higher ratio of Δ5 to Δ4 steroids (P, 17OH-P and androstendione). However, 17OH-P concentrations may be elevated as a result of peripheral conversion of Δ5-17OH-pregnenolone by the 3βHSD type 1 enzyme. Biochemical diagnosis is based on the finding of elevated concentrations of 17OH-pregnenolone (>150 nmol/L) at baseline or after ACTH stimulation [58]. Molecular diagnosis will confirm the presence of *HSD3B2* inactivating mutations in homozygosis or compound heterozygosis (Table 2).

The enzyme 11β-hydroxylase converts 11-deoxicortisol into cortisol and 11-deoxycorticosterone into corticosterone (Figure 2). 11β-hydroxylase deficiency induces CAH with concurrent cortisol and aldosterone deficiency resulting in androgen synthesis (T), and it is associated with a very significant virilization of the female fetus. The hormone profile is characterized by diminished levels of cortisol and aldosterone, with increased ACTH concentrations but inhibited PRA. The most robust diagnostic marker is elevated levels of 11-deoxicortisol and 11-deoxicorticosterone, although determination of these parameters is not available in many clinical laboratories. Up to 60% of patients have hypertension secondary to 11-deoxicorticosterone accumulation, which has mineralocorticoid activity. The steroid profile in urine shows reduced levels of cortisol and increased levels of 11-deoxicorticosterone metabolites [59].

There are mild forms of the enzyme deficiency [60]. Molecular diagnosis will confirm the presence of mutations in the *CYP11B1* gene in homozygosis or compound heterozygosis (Table 2).

Resistance to glucocorticoids is a very rare cause of virilization of a female fetus. A mutation of the glucocorticoid receptor gene (*GRα* or *NR3C1*) (Table 2) causes cortisol and ACTH hypersecretion, without clinical evidence of hypercortisolism but with manifestations of androgen and mineralocorticoid excess [61].

Estrogen resistance induced by inactivating mutations in the E2 receptor alpha (*ESR1* gene) (Table 2) is a very rare condition that was first described in males; in females, the morphology of external genitalia at birth has not yet been described in detail, but patients develop postnatal virilization by the development of a polycystic ovary with increased levels of androstenedione and T, as well as tall stature and osteoporosis [62].

#### b) Increased fetoplacental androgen production

P450-oxidoreductase (POR) is a flavoprotein bound to the membrane of cytochrome *c* that plays a crucial role in electron transfer from NADPH to microsomal enzymes P450 (CYP21, CYP17, and CYP19 or aromatase). POR deficiency is characterized by a partial, varying impairment of different enzyme activities (Figure 2): 17α-hydroxylase and 17,20-lyase, associated or not with 21-hydroxylase and aromatase. Patients exhibit a broad phenotypical spectrum and may present characteristic skeletal malformations (Antley–Bixler syndrome). It may cause CAH and genital ambiguity [63]. As to biochemistry, patients may have normal or low levels of cortisol, high levels of 17OH-P and T, and abnormal concentrations of some steroids (and their metabolites) of the backdoor pathway (Figure 3). The steroid profile in urine shows accumulation of pregnenolone and P metabolites. Female neonates have ambiguous genitalia due to fetal exposure to excess androgens secondary to aromatase (CYP19) deficiency and/or DHT synthesis through the backdoor pathway [64]. The mother may show signs of virilization during pregnancy, with elevated T concentrations. Virilization of the female neonate does not progress, and circulating androgen concentrations remain normal until puberty, when ovarian E2 synthesis decreases and the production of backdoor pathway steroidogenesis precursors increases [65]. Molecular diagnosis will confirm the presence of *POR*-inactivating mutations in homozygosis or compound heterozygosis (Table 2).

The enzyme aromatase (CYP19) catalyzes T conversion into E2 and DHEA conversion into estrone (E1). In the absence of aromatase activity, the placenta cannot convert DHEA sulphate, produced in large amounts by the fetal adrenal gland, into estrogens (E1, E2, and estriol) and transforms it into T, which causes virilization of the 46,XX fetus and the mother [66]. Patients generally show elevated levels of T and gonadotropins, especially FSH. Molecular diagnosis will confirm the presence of mutations in the *CYP19A1* gene in homozygosis or compound heterozygosis (Table 2).

Androgen-producing fetal or placental tumors: cases have been reported of congenital adrenal tumors causing the virilization of a 46,XX fetus.

#### c) Elevation of maternal androgen production

Excess androgens may be transferred from the mother to the 46,XX fetus, either by secretion from virilizing tumors during pregnancy (including pregnancy luteoma and Krukenberg tumor), by pharmacological therapies or by environmental pollutants with androgenic effects, or by poor therapeutic monitoring during pregnancy of a mother with CAH (Table 1).

### 3) Internal genitalia development disorder

Some individuals may exhibit isolated malformations of female internal genital ducts (uterus, vagina, and fallopian tubes). These malformations may be caused by incomplete development or the presence of abnormal structures (Table 1). They are not frequent and, in some cases, there may be a family history of malformations, which suggests a genetic origin, although the etiology is rarely elucidated.

There are no specific biochemical markers. Only amenorrhea, dysmenorrhea, and infertility may give some diagnostic guidance. Malformations include aplasia or hypoplasia of the uterus and the fallopian tubes, a bicornuate or bipartite uterus that may be associated with malformations in other systems or tissues, such as the hand–foot–genital syndrome (associated with the *HOXA13* gene); MURCS syndrome (Mullerian aplasia, renal aplasia, cervico–thoracic somite abnormalities, currently defined as multigenic); and the MRKH (Mayer–Rokitansky–Kuster–Hauser) syndrome types I and II, where several genetic disorders have been described; and finally *WNT4*-inactivating mutations, which have been associated with ovotesticular or testicular development with potential Mullerian duct aplasia (Table 2).
